# Identification and Characterization of *Crr1a*, a Gene for Resistance to Clubroot Disease (*Plasmodiophora brassicae* Woronin) in *Brassica rapa* L.

**DOI:** 10.1371/journal.pone.0054745

**Published:** 2013-01-30

**Authors:** Katsunori Hatakeyama, Keita Suwabe, Rubens Norio Tomita, Takeyuki Kato, Tsukasa Nunome, Hiroyuki Fukuoka, Satoru Matsumoto

**Affiliations:** 1 Vegetable Breeding and Genome Research Division, NARO Institute of Vegetable and Tea Science, Tsu, Mie, Japan; 2 Graduate School of Bioresources, Mie University, Tsu, Mie, Japan; National Taiwan University, Taiwan

## Abstract

Clubroot disease, caused by the obligate biotrophic protist *Plasmodiophora brassicae* Woronin, is one of the most economically important diseases of *Brassica* crops in the world. Although many clubroot resistance (CR) loci have been identified through genetic analysis and QTL mapping, the molecular mechanisms of defense responses against *P. brassicae* remain unknown. Fine mapping of the *Crr1* locus, which was originally identified as a single locus, revealed that it comprises two gene loci, *Crr1a* and *Crr1b*. Here we report the map-based cloning and characterization of *Crr1a*, which confers resistance to clubroot in *Brassica rapa*. *Crr1a^G004^*, cloned from the resistant line G004, encodes a Toll-Interleukin-1 receptor/nucleotide-binding site/leucine-rich repeat (TIR-NB-LRR) protein expressed in the stele and cortex of hypocotyl and roots, where secondary infection of the pathogen occurs, but not in root hairs, where primary infection occurs. Gain-of-function analysis proved that *Crr1a^G004^* alone conferred resistance to isolate Ano-01 in susceptible Arabidopsis and *B. rapa*. In comparison, the susceptible allele *Crr1a^A9709^* encodes a truncated NB-LRR protein, which lacked more than half of the TIR domain on account of the insertion of a solo-long terminal repeat (LTR) in exon 1 and included several substitutions and insertion-deletions in the LRR domain. This study provides a basis for further molecular analysis of defense mechanisms against *P. brassicae* and will contribute to the breeding of resistant cultivars of *Brassica* vegetables by marker-assisted selection.

Data deposition The sequence reported in this paper has been deposited in the GenBank database (accession no. AB605024).

## Introduction

Clubroot is one of the most serious diseases of cruciferous crops in the world. *Plasmodiophora brassicae*, the causal agent of this disease, is not a fungus but a member of the phylum Cercozoa in the kingdom Rhizaria [1], [2], [3]. The first record of clubroot in Japan dates from 1892 in cabbage and 1898 in Chinese cabbage [4], and this disease is now considered a major problem in cabbage and Chinese cabbage production in Japan and Korea [5]. The life cycle of *P. brassicae* is divided into two phases: a primary phase occurring in the root hairs and a secondary phase occurring in the stele and cortex of the hypocotyl and roots [4]. During the secondary phase, secondary plasmodia induce abnormal tissue proliferation of infected roots, leading to the formation of galls (clubs). These symptoms prevent the uptake of water and nutrients, stunting the infected plants and severely reducing crop yield and quality [6]. The primary phase has been observed in both susceptible and resistant plants. In the secondary phase, the development of the plasmodia is quantitatively reduced or delayed in resistant plants [7], [8], [9]. Because the resting spores released from decayed clubs can survive for many years in soil, agricultural practices such as liming and crop rotation are insufficient to keep crops healthy. In addition, reducing the use of agrochemicals is preferred for the production of vegetables. Therefore, the breeding of resistant cultivars is one of the most efficient ways to control clubroot.

European fodder turnips (*Brassica rapa*) were identified as sources of resistance and have been used to transfer their clubroot resistance (CR) genes into Chinese cabbage, oilseed rape, and *Brassica oleracea* [5], [10]. The CR trait of European turnip cultivars such as Siloga, Gelria R, and Debra was considered at first to be controlled by a single dominant gene [11]. Yet after the release of more than 50 CR F_1_ cultivars of Chinese cabbage so far, the breakdown of resistance, caused by variation in the pathogenicity of *P. brassicae*, has been reported only recently [12], [13]. Several differential tester sets have been used to study the interaction between *P. brassicae* and hosts [12], [13], [14], [15], [16]. Four pathotypes (groups 1 to 4) were identified in Japanese field isolates through the use of two commercial CR F_1_ cultivars of Chinese cabbage [12], [13]. But since the number and identity of resistance genes in the tester sets are unknown [10], information on the performance or pathotype specificity of CR genes remains limited.

Genetic analysis and quantitative trait locus (QTL) mapping studies have identified at least 8 CR loci in *B. rapa*, 22 QTLs in *B. oleracea*, and 16 QTLs in *Brassica napus* [10], [17]. In *B. rapa*, *Crr1* and *Crr2* were identified on chromosomes A08 and A01, respectively [18], [19]. These two loci were detected by using two *P. brassicae* isolates, the mild Ano-01 and the more virulent Wakayama-01. *Crr1* was necessary for the resistance to both isolates, but plants having *Crr1* alone were susceptible to Wakayama-01. *Crr2*, which by itself does not show any effect against either isolate, was necessary for resistance to Wakayama-01 in combination with *Crr1*. Therefore, *Crr1* may play a role in a common pathway of resistance, and *Crr2* may be a modifier locus for the resistance expressed by *Crr1* [19]. Four CR loci–*CRa* [20], [21], [22], *CRb* [23], *Crr3* [24], [25], and *CRk* [26]–were identified on chromosome A03. The relationships between common markers and the location of the CR loci suggest that *CRa* and *Crr3* are identical, allelic, or closely linked to *CRb* and *CRk*, respectively [10]. *CRc* [26] was mapped on A02, and a weak QTL, *Crr4* [19], on A06. Comparative mapping of CR loci between *B. rapa* and Arabidopsis revealed that *Crr1*, *Crr2*, and *CRb* are syntenic with the central region of Arabidopsis chromosome 4 [19], [25]. Because this region is located within a disease resistance gene cluster, it has been suggested that CR genes are members of these clusters [17], [19]. However, although many studies have mapped CR loci in *B. rapa*, the molecular mechanisms of resistance remain unknown.

In a previous study, we fine-mapped the *Crr1* locus and found that it was likely to comprise two gene loci, a major locus for clubroot resistance and another locus with minor effect [3]. We named the former locus *Crr1a* and the latter *Crr1b*. Here, we report the map-based cloning and characterization of *Crr1a*, the major locus for resistance to Ano-01, derived from European fodder turnip ‘Siloga’. *Crr1a^G004^* encodes a TIR-NB-LRR disease resistance protein and is expressed in the stele or cortex of hypocotyl and roots, where the secondary infection phase occurs. Transgenic Arabidopsis and susceptible *B. rapa* harboring *Crr1^G004^* showed resistance to *P. brassicae* isolates similar to that of the resistant *B. rapa*.

## Results

### Map-based Cloning of Crr1a

**Figure 1:**
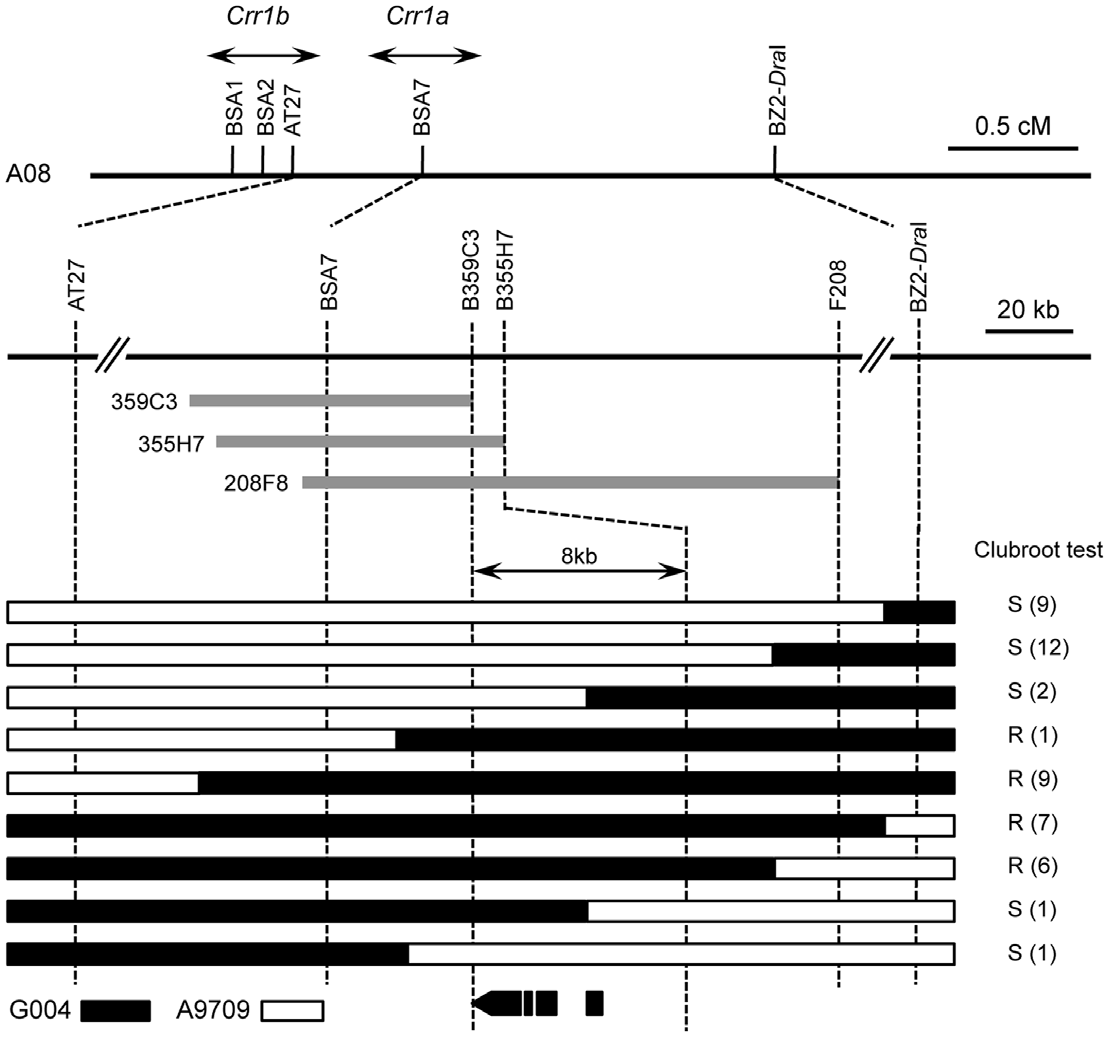
Map-based cloning of *Crr1a*: a genetic map around the *Crr1* locus and graphical genotypes of F_3_ populations in which recombination occurred between AT27 and BZ2–*Dra*I. The major locus for resistance to Ano-01 was predicted around BSA7, and another locus with minor effect was predicted around BSA2 (Suwabe et al., 2011). We named the former locus *Crr1a* and the latter *Crr1b*. Gray bars indicate BAC clones isolated by using BSA7 as a probe. Black bars, homozygous for resistant G004 allele; white bars, homozygous for susceptible A9709 allele. Phenotypes of resistance (R) and susceptibility (S) of F_3_ populations to Ano-01 are indicated to the right of the graphical genotype, with the number of F_3_ populations of each in parentheses. The 3 F_3_ populations in which recombination occurred between B359C3 and B355H7 were susceptible to Ano-01. The position of *Crr1a* was delimited to an 8-kb region between B355H7 and B359C3. The predicted ORFs of the *Crr1a* candidate are shown below.

We previously fine-mapped the *Crr1* locus by analyzing 1920 F_2_ plants derived from a cross between clubroot-resistant G004 and susceptible A9709 and found that *Crr1* was likely to consist of two gene loci in the region around insertion–deletion (indel) markers BSA2 and BSA7 [3]. We named the major locus for resistance, located near BSA7, *Crr1a*, and the other locus, with minor effect, around indel marker BSA2, *Crr1b* (Fig. 1). Here we attempted to delimit the candidate region of the *Crr1a* locus. A BAC library was screened with BSA7, and three BAC-end markers were developed (Fig. 1). We genotyped these markers in the F_2_ population of 3700 plants and found 39 F_2_ plants with recombination in the region between BSA7 and BZ2–*Dra*I. A clubroot resistance test using F_3_ seeds derived from the F_2_ plants revealed that three F_3_ populations obtained from F_2_ plants in which recombination occurred between markers B355H7 and B359C3 were susceptible to isolate Ano-01 (Fig. 1). Therefore, *Crr1a* was estimated to lie within this 8-kb region. We determined the sequence of BAC clone 208F8. 5′-RACE (rapid amplification of cDNA ends) and 3′-RACE experiments revealed that four open reading frames (ORFs) predicted in this region formed a single gene with similarity to a TIR-NB-LRR–type *R* (resistance) gene.

**Figure 2:**
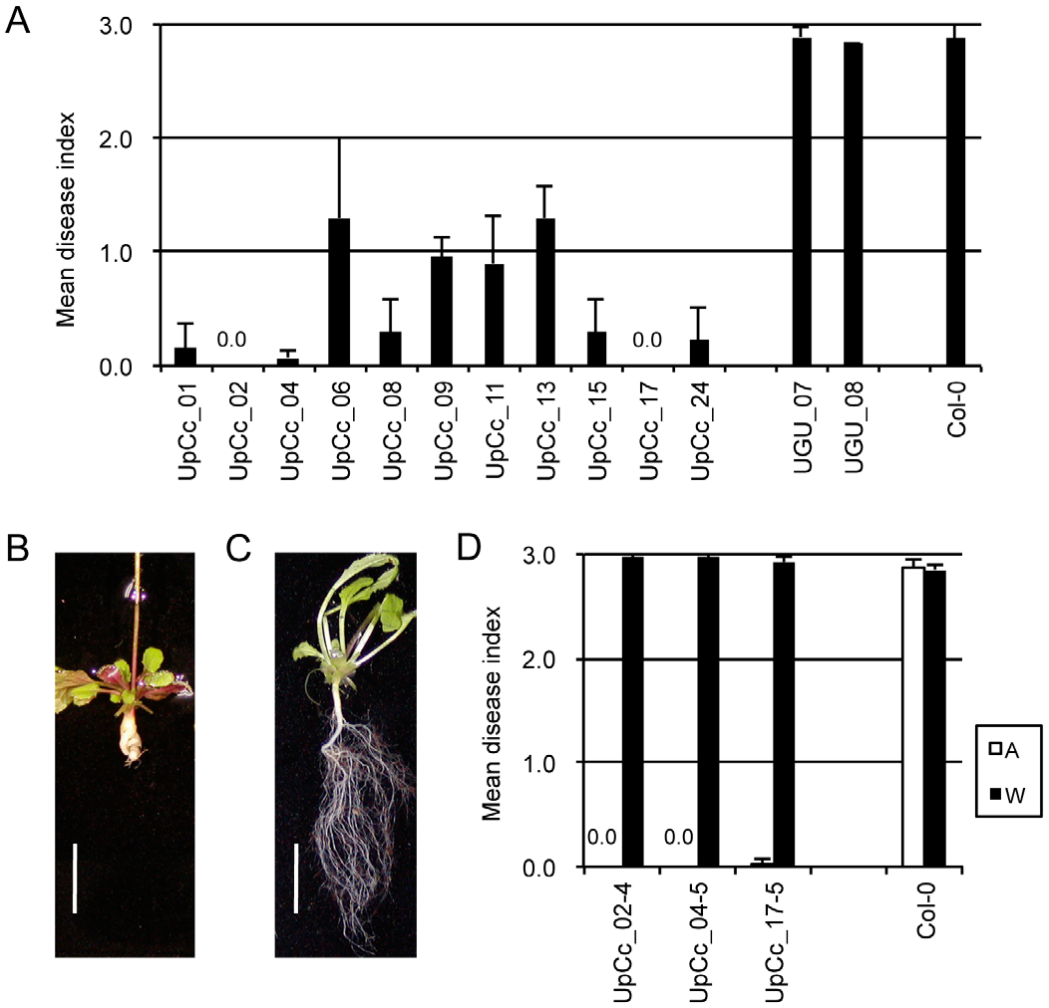
Complementation test of *Crr1a* candidate. A. Resistance of transgenic T_2_ lines (UpCc_01–24), *LsUbi* promoter::*GUS* lines (UGU_07, 08), and wild-type Col-0 to Ano-01. Means (±SD) are based on the average of 2 or 3 clubroot tests (9 plants per test). A lower mean disease index score indicates higher resistance to clubroot. B, C. Root phenotypes of (B) Col-0 inoculated with Ano-01 and (C) transgenic T_2_ line inoculated with Ano-01. Scale bar indicates 10 mm. D. Resistance of T_3_ lines to Ano-01 (A) and Wakayama-01 (W).

To determine whether this candidate gene confers resistance to *P. brassicae*, we connected a full-length cDNA of *Crr1a^G004^* cloned by RT-PCR with the *Lactuca sativa Ubiquitin* (*LsUbi*) promoter and transferred it into clubroot-susceptible Arabidopsis Col-0. The *Crr1* locus was effective against Ano-01 but not against the more virulent Wakayama-01 [18]. Therefore, we tested T_2_ seeds derived from 11 independent transformants (T_1_) for resistance to Ano-01 and Wakayama-01. Col-0 plants and *LsUbi* promoter::*GUS* lines (UGU_07, 08) were fully susceptible to Ano-01 (Fig. 2A, B). Nine T_1_ lines were highly resistant to Ano-01 (mean disease index [DI] <1.0) (Fig. 2C) and 2 were intermediate (mean DI >1.0). In contrast, 7 T_1_ lines showing resistance to Ano-01 were fully susceptible (DI >2.6) to Wakayama-01 (data not shown). Three T_3_ lines derived from resistant T_2_ plants homozygous for the *Crr1a^G004^* transgene (UpCc_02, UpCc_04, UpCc_17) were also resistant to Ano-01 and susceptible to Wakayama-01 (Fig. 2D). For further verification, we expressed the full-length cDNA of *Crr1a^G004^* under the control of its own 2.5-kb upstream region; all 11 T_2_ lines tested were highly resistant to Ano-01 (Fig. S1).

Previously, using two CR F_1_ cultivars of Chinese cabbage–CR Ryutoku and SCR Hiroki–as differential hosts, we classified Japanese field isolates into four pathotypes: group 1, which was virulent on both cultivars; group 2, which was virulent only on CR Ryutoku; group 3, which was virulent only on SCR Hiroki; and group 4, which was not virulent on either [13]. Ano-01 was classified into group 4, while Wakayama-01 was classified between groups 1 and 2 because of an intermediate response by SCR Hiroki. We inoculated two transgenic T_2_ lines homozygous for the *Crr1a^G004^* transgene with a representative isolate of each pathotype and found that *Crr1a^G004^* was effective against groups 2 and 4, but not against groups 1 and 3 (Fig. S2). These results were similar to those of *B. rapa* with a homozygous *Crr1* locus. Thus, these results strongly indicate that this candidate gene confers resistance to *P. brassicae* isolates and is *Crr1a*.

### *Crr1a* Encodes a TIR-NB-LRR–type Disease Resistance Protein

**Figure 3:**
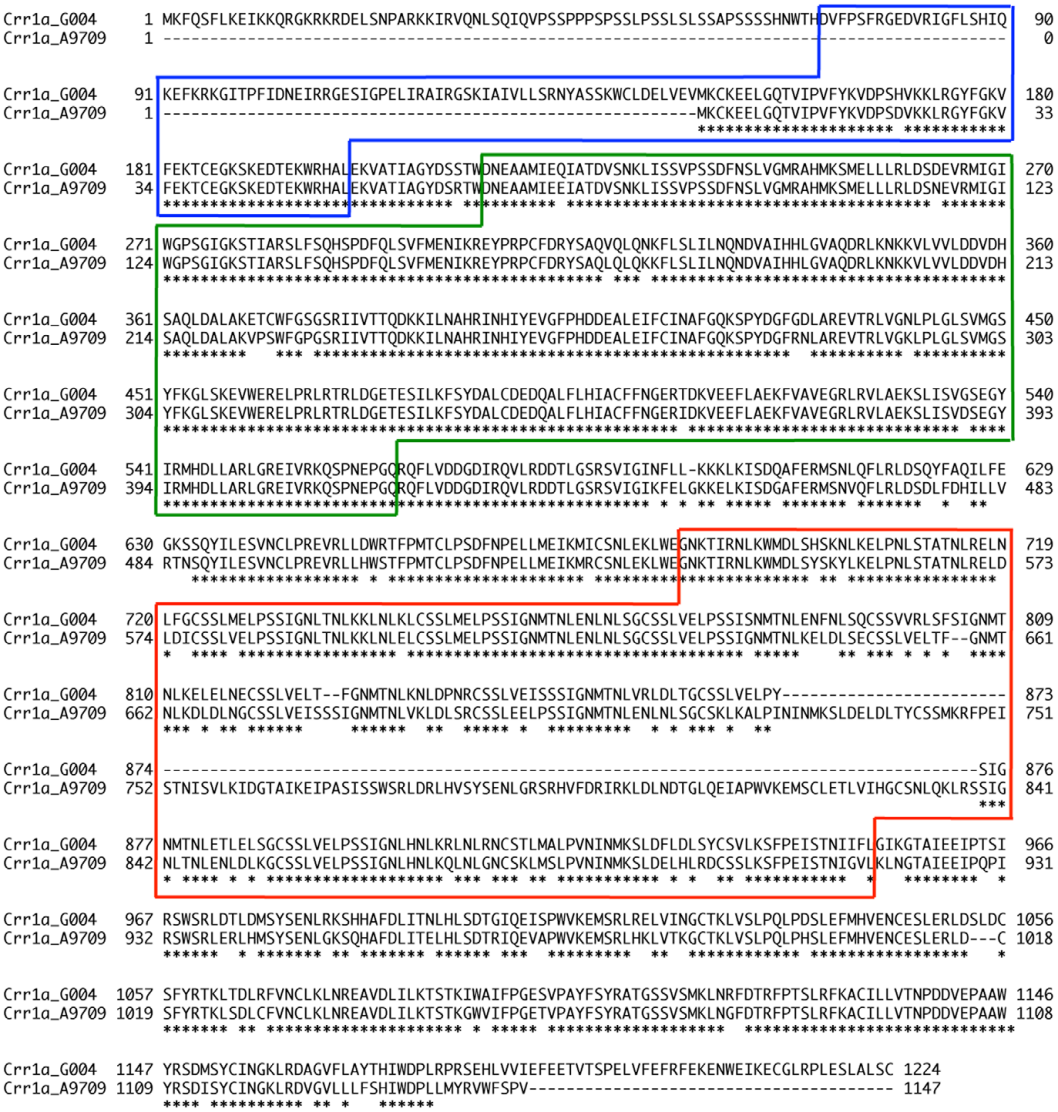
Alignment of deduced amino acid sequences of *Crr1a* between resistant G004 and susceptible A9709 alleles. Asterisks, identical amino acid residues; dashed lines, gaps for alignment. Blue, TIR domain; green, NB domain; red, LRR domain.

Comparison of the genomic DNA and cDNA sequences revealed that *Crr1a^G004^* consists of 4 exons and 3 introns. *Crr1a^G004^* encodes a putative 1225-aa protein consisting of an N-terminal Toll-Interleukin-1 receptor (TIR) domain, a nucleotide-binding site (NB) domain, and leucine-rich repeats (LRRs) at the C-terminus of the NB domain (Fig. 3). Conserved motifs characteristic of the NB domain–the P-loop, kinase-2, RNBS-B, GLPL, and MHDV domains–were also identified [27]. In the LRR region, at least 11 imperfect repeats with lengths of 22 to 24 aa were identified. A BLAST search revealed that the predicted Crr1^G004^ protein showed similarity to At5g11250 (58% identity), which is a TIR-NB-LRR–type *R* gene in the TNL-G subgroup [27]. Among known *R* genes, the predicted Crr1a^G004^ protein showed moderate similarity to Arabidopsis *RPP1*s (55% identity), which confer resistance to the biotrophic oomycete *Peronospora parasitica* (downy mildew) [28].

### Structural Differences between Resistant and Susceptible Alleles

**Figure 4:**
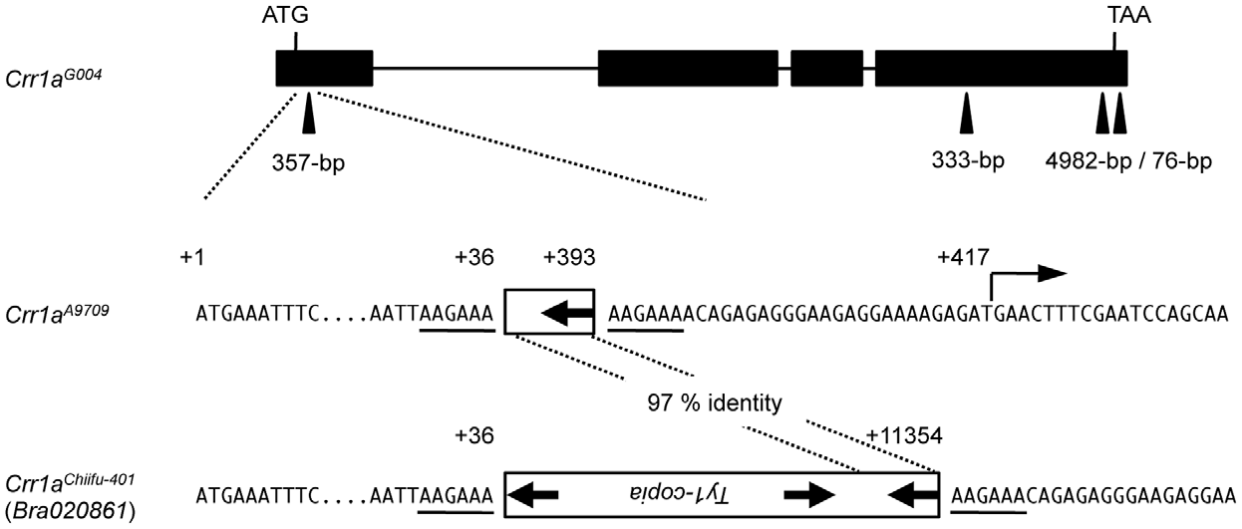
Sequence polymorphisms of *Crr1a* between resistant and susceptible alleles. *Crr1a^G004^*: Schematic representation of *Crr1a* allelic structure in resistant G004. Black boxes, exons; black lines, introns; arrowheads, large insertions. *Crr1a^A9709^*: Sequence surrounding the site of the 357-bp insertion in susceptible A9707. White box, retrotransposon-like sequence. Bent arrow, transcription start site of *Crr1a^A9709^* predicted by 2 independent 5′-RACE analyses. Putative target site duplication is underlined. *Crr1a^Chiifu-401^*: Corresponding sequence from susceptible Chiifu-401. The 357-bp insertion in *Crr1a^A9709^* was almost identical to the 3′-terminal region of a 4546-bp *copia*-like retrotransposon found in *Crr1a^Chiifu-401^*.

We determined the nucleotide sequence between markers B355H7 and B359C3 in the susceptible A9709 (Fig. 1) and compared it with that in the resistant G004. *Crr1a^A9709^* had three large insertions: a 357-bp insertion 37 bp downstream of the start codon, and 333- and 4982-bp insertions in exon 4 (Fig. 4). The 4982-bp insertion, 157 bp upstream of the termination codon of *Crr1a^G004^*, showed high similarity to a *Ty1*-*copia* long terminal repeat (LTR) retrotransposon comprising two identical 171-bp LTRs bordered by 6 bp of target site duplication (TSD) (Fig. S3). Interestingly, 5′- and 3′-RACE analysis placed the 5′ end of *Crr1a^A9709^* 25 bp downstream of the 3′ end of the 357-bp insertion (Fig. 4) and the 3′ end in the first LTR region of the retrotransposon (Fig. S3). Translation from the first available in-frame start codon would yield an NB-LRR protein lacking 80 residues of the TIR domain and 49 residues of the C-terminal domain (Fig. 3). Comparison of the predicted proteins of both alleles showed that the remaining TIR and NB domains were well conserved, but the LRR domain was highly variable (56% identity; Fig. 3). The LRR domain of the predicted Crr1a^A9709^ protein included 50 substitutions, 2 deletions, and insertions of 2 and 111 residues relative to the predicted Crr1a^G004^ protein.

We found a highly homologous sequence (*Bra020861*) in the reference *B. rapa* genome sequence derived from a clubroot-susceptible line, Chiifu-401 [29], and considered that it is an allele of *Crr1a*. Comparison of the nucleotide sequences between *Crr1a^A9709^* and *Crr1a^Chiifu-401^* revealed that the sequences between markers B355H7 and B359C3 were almost identical except in the insertion in exon 1. Interestingly, large insertions in exons 1 and 4 were also found in *Crr1a^Chiifu-401^*, both with high similarity to the LTR retrotransposon (Fig. 4, Fig. S3). Although the sequence similarity and domain order suggest that the insertion in exon 1 of *Crr1a^Chiifu-401^* is a *Ty1-copia* LTR retrotransposon, it is not probably an intact retrotransposon, because it has no start codon and it has an inverted repeat of LTRs at the 5′-end. Because the 357-bp insertion in *Crr1a^A9709^* showed high similarity to the LTR region at the 5′-end of the insertion in *Crr1a^Chiifu-401^* and TSDs were well conserved between both alleles (Fig. 4), it is likely that the 357-bp insertion is a solo-LTR.

### Expression Analysis of Crr1^G004^

**Figure 5:**
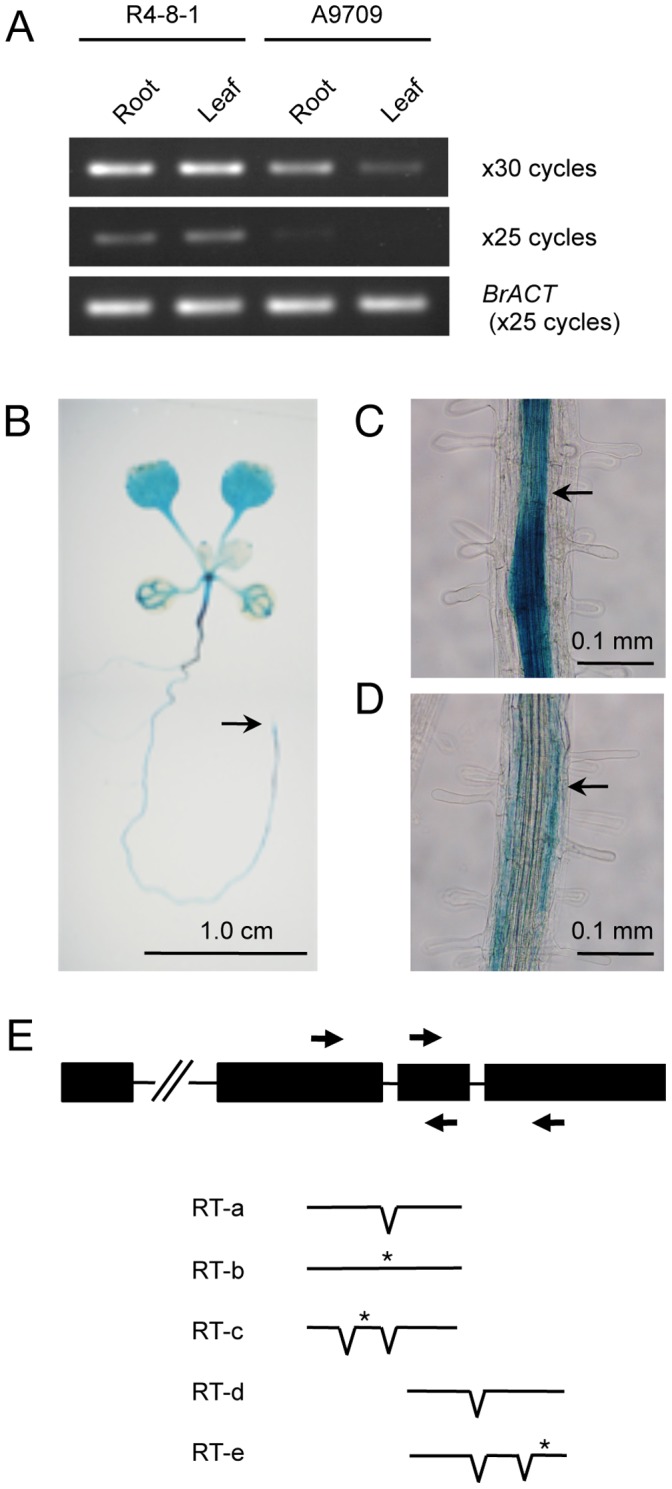
Expression analysis of *Crr1a*. A. Semi-quantitative RT-PCR analysis of *Crr1* in resistant (R4-8-1) and susceptible (A9709) lines. The *B. rapa* actin gene (*BrACT*) was used as a control. PCR cycles are indicated on the right. B-D. Spatial expression of *Crr1^G004^* shown as GUS staining in (B) 12-day-old seedlings of *Crr1^G004^* promoter::*GUS* plants (arrow indicates extreme end of primary root), (C) stele (arrow) of primary root, and (D) cortex (arrow) of primary root. E. Schematic diagram of gene structure of *Crr1a^G004^* and alternative transcripts obtained by RT-PCR using primers flanking introns 2 (RT-a, -b, and -c) and 3 (RT-d and -e). V-like lines, spliced introns; asterisks, predicted stop codons.

Semi-quantitative RT-PCR revealed *Crr1a^G004^* transcripts in roots and leaves of the resistant R4-8-1 (see “Experimental procedures”) and the susceptible A9709, more so in the former (Fig. 5A). We examined the expression of *Crr1a^G004^* in transgenic Arabidopsis carrying a *Crr1a^G004^* promoter::*GUS* reporter gene construct. In 12-day-old seedlings, the construct was expressed in the stele and cortex of the primary root and hypocotyl, the vascular bundles of the cotyledons and leaves, and at the center of rosettes in a region corresponding to the shoot apical meristem (Fig. 5B–D). No expression was detected in root hairs, the root cap, or the adjacent elongation zone (Fig. 5B–D).

In the course of cloning the full-length cDNAs by RT-PCR, we obtained two clones, each containing a cryptic intron, one in exon 2 and the other in exon 4. This result indicates that *Crr1a^G004^* is alternatively spliced like other TIR-NB-LRR–type *R* genes [30], [31]. RT-PCR with primers flanking introns 2 and 3 produced faint bands in addition to the major band. We cloned and sequenced the PCR products and, in addition to the regular transcript with introns spliced out (RT-a and RT-d in Fig. 5E), obtained a longer transcript with a retained intron 2 (RT-b). In addition, the shorter PCR product obtained with primers flanking introns 2 and 3 represented transcripts in which a cryptic intron within exon 2 or exon 4 was spliced out (RT-c and RT-e, respectively). No PCR products with retained introns 1 and 3 were obtained. Because the retained and cryptic introns gave rise to in-frame stop codons or a frame shift, all three alternative transcripts encode truncated TIR-NB proteins.

### Characterization of Transgenic B. rapa Plants Expressing Crr1a cDNA

**Figure 6:**
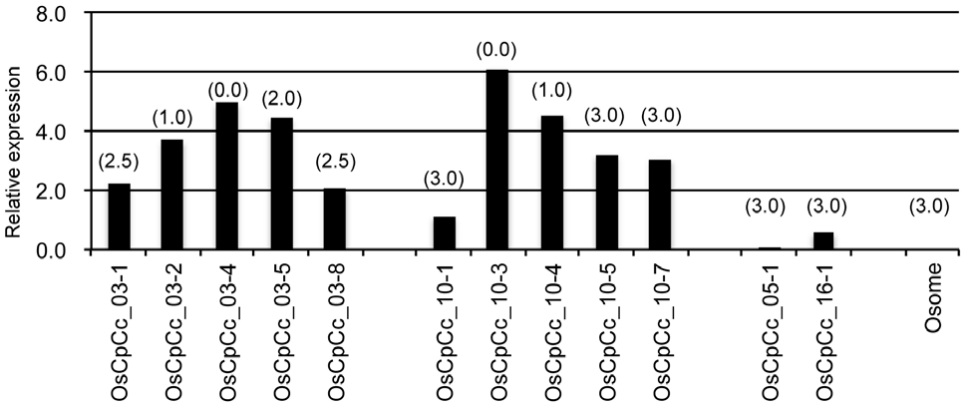
Expression of the *Crr1a^G004^* transgene in T_1_ lines inoculated with Ano-01. Transcript levels of 5 representative T_1_ plants derived from each of 2 resistant T_0_ lines and 1 plant derived from each of 2 susceptible T_0_ lines are shown. Osome is the recipient. The disease index of each plant is indicated in parentheses above each bar. Similar results were obtained in 2 independent tests.

To assess the ability of the *Crr1a^G004^* allele to confer resistance in susceptible *B. rapa*, we inserted the *Crr1a^G004^* promoter::*Crr1a^G004^* cDNA construct into a susceptible cultivar of *B. rapa*. T_1_ seeds of 15 independent transgenic plants (T_0_) were tested for resistance to Ano-01. Five of the T_0_ lines produced resistant T_1_ plants (DI = 0 or 1; Table 1, Fig. S4). Other T_1_ plants with the transgene showed severe (DI = 3.0) or moderate swelling (DI = 2.5) on the main roots. Because *Crr1a^G004^* was expressed in both roots and leaves, we analyzed the expression of the transgene in leaves of resistant and susceptible T_1_ plants derived from two resistant T_0_ lines. Expression of the transgene in the resistant plants was 2 to 3 times that in the susceptible plants (Fig. 6). Similar results were observed in 2 independent tests. These results indicate that *Crr1a^G004^* functions in susceptible *B. rapa* and dose-dependently confers resistance to Ano-01.

**Table 1:** Clubroot resistance of the transgenic *Brassica rapa* (T_1_) carrying *Crr1a* promoter::*Crr1a* cDNA construct.

	Disease index (DI)						
Cultivar/Line (T_0_)	0	1	2	2.5	3	No.plants tested	mean DI
Osome	0	0	0	0	16	16	3.0
OsCpCc_03	0	12	5	11	13	41	2.2
OsCpCc_04	0	0	0	2	30	32	3.0
OsCpCc_05	0	0	0	1	41	42	3.0
OsCpCc_07	0	0	1	9	13	23	2.8
OsCpCc_08	0	5	6	10	12	33	2.4
OsCpCc_09	0	0	0	5	21	26	2.9
OsCpCc_10	2	11	5	4	8	30	1.8
OsCpCc_12	0	2	0	4	9	15	2.6
OsCpCc_15	0	7	8	4	9	28	2.1
OsCpCc_16	0	0	0	0	20	20	3.0
OsCpCc_18	0	0	0	0	10	10	3.0
OsCpCc_19	0	0	0	3	12	15	2.9
OsCpCc_20	0	0	1	1	10	12	2.9
OsCpCc_21	0	0	0	0	12	12	3.0
OsCpCc_22	0	0	0	0	4	4	3.0

Root symptoms of each disease index are shown in Fig. S1.

## Discussion

The *Crr1* locus was originally identified as a single locus for resistance to clubroot isolate Ano-01 [19]. Fine mapping of this locus revealed two gene loci [3], *Crr1a*, with a major effect, and *Crr1b*, with a minor effect. We have now cloned and characterized *Crr1a^G004^*. Recent findings show that a pair of NB-LRR genes function together in disease resistance [32]. In contrast, our finding that *Crr1a^G004^* alone could confer resistance to Ano-01 in transgenic Arabidopsis and *B. rapa* suggests that *Crr1b* is not required for *Crr1a^G004^*-mediated resistance.

*Crr1a^G004^* encodes the TIR-NB-LRR class of R protein (Fig. 3), which confers resistance to viral, fungal, and oomycete pathogens. Resistance to the obligate biotroph protist *P. brassicae* also might be mediated via a gene-for-gene interaction, called effector-triggered immunity [33]. Expression of *Crr1a^G004^* was detected in the stele and cortex of hypocotyl and root, where secondary infection occurs, but not in root hairs, where primary infection occurs (Fig. 5B–D). These results are consistent with previous histological findings of the incompatible interaction [7], [8], [9], [10]. Because primary infection occurs in both resistant and susceptible plants [10], this phase is not likely to be associated with resistance. Tanaka et al. [9] reported that the plasmodia remained immature with a small number of nuclei and did not form resting spores even by 40 days after inoculation of Kukai 70, a CR cultivar of Chinese cabbage. Similar findings were also reported in resistant radish cultivars and accessions of Arabidopsis [7], [8], [9]. Therefore, Crr1a^G004^ may inhibit plasmodial development during the secondary infection phase.

Gain-of-function analysis proved that *Crr1a^G004^* alone confers resistance to Ano-01 in susceptible Arabidopsis and *B. rapa*. Resistant and susceptible T_1_ plants of *B. rapa* segregated, with the level of resistance dependent on the expression level of the transgene (Fig. 6). It is likely that T_1_ plants homozygous for the transgene were resistant and plants heterozygous were susceptible. Our unpublished data suggest that *Crr1* is incompletely dominant, because genetic analysis of an F_2_ population derived from a cross between G004 and A9709 showed that a heterozygous *Crr1* locus was insufficient for complete resistance to Ano-01. Our results here suggest that a threshold level of *Crr1a* expression is required for complete resistance, and the level of expression may explain the incomplete dominance of *Crr1*. However, we cannot rule out the possibility that a low level of susceptible Crr1a protein acts as a dominant-negative regulator of Crr1a^G004^ in heterozygous plants. In the case of the tobacco *N* gene, a member of the TIR-NB-LRR class of *R* genes, loss-of-function alleles caused by TIR deletion or point mutations in TIR interfered with the wild-type N function in heterozygous plants [34]. In Arabidopsis, all T_2_ plants with the transgene showed resistance, and *Crr1a^G004^* behaved as a dominant resistance gene (Fig. 2). This different behavior may be due to the difference in inoculation methods between Arabidopsis and *B. rapa.* The level of expression of the transgene in heterozygous plants might be high enough to confer resistance in to lower concentration of resting spores.

The genomic regions of *B. rapa* adjacent to *Crr1*, *Crr2*, and *CRb* are syntenic with Arabidopsis chromosome 4 [19], [23], [25]. Therefore, these CR loci may have been derived from the same region of the ancestral genome, and triplicated and dispersed among 3 chromosomes in *B. rapa* during the evolution of the *Brassica* genome [5]. The genomic region around *Crr3* is syntenic with Arabidopsis chromosome 3, and this locus is thought to have a different origin from *Crr1*, *Crr2*, and *CRb* [25]. Here, however, we found that *Crr1a^G004^* showed the highest similarity to At5g11250 on Arabidopsis chromosome 5. Because two additive QTLs controlling partial resistance to clubroot in Arabidopsis accession Bur-0 have been identified on chromosome 5 and one of them co-localized with several clusters of resistance genes [35], either locus might be a functional ortholog of *Crr1a* and the ancestor of CR genes. Sequence information on *Crr1a^G004^* will accelerate the cloning of other CR genes identified in *Brassica* and Arabidopsis, and may clarify the evolution of CR genes. In fact, a BLASTP search of the *B. rapa* genome sequence revealed a large number of predicted proteins with partial similarity to Crr1a^G004^, some near markers linked to other, previously reported CR loci (data not shown).

The susceptible *Crr1^A9709^* allele had the solo-LTR in exon 1 and appeared to generate a truncated NB-LRR protein lacking more than half of the TIR domain (Fig. 3). The TIR domain of the NB-LRR protein plays an important role in the induction of defense responses or in recognition specificity for the pathogen effector [36], [37], [38]. Therefore, it is likely that deletion of the TIR domain abolishes the Crr1a^A9709^ function and results in susceptibility. The large LTR retrotransposon-like insertion in exon 1 was also found in the susceptible *Crr1a^Chiifu-401^* allele, and the insertion site was conserved between *Crr1a^A9709^* and *Crr1a^Chiifu-401^*. These results suggest that both susceptible alleles are derived from the same retrotransposon insertion event, which causes loss of resistance, and that this retrotransposon has a role in the differentiation of CR genes. However, this insertion is not the sole cause of susceptibility alleles in *B. rapa*, because PCR analysis using primers flanking this insertion revealed that 18 of 24 non-CR cultivars of Chinese cabbage and turnips tested did not have such an insertion (data not shown). Furthermore, since the LRR domain plays an important role in recognition specificity [37], we cannot exclude the possibility that indels or substitutions in the LRR domain result in susceptibility.

*Crr1^G004^* was alternatively spliced (Fig. 5E). Alternative splicing is a common feature of NB-LRR–type *R* genes. Although its biological role in the function of *R* genes is still unknown, transcript variants of tobacco *N* and Arabidopsis *RPS4* are required for complete resistance, and the expression of the alternative transcripts is induced during defense responses [30], [31]. Our transgenic plants with the full-length *Crr1* cDNA, which did not produce truncated transcripts by alternative splicing, were resistant to Ano-01 (Fig. 2, 6); this result suggests that transcript variants are not necessary for Crr1^G004^ function. A similar finding was also reported in relation to the flax rust resistance gene *L^6^* [39].

An effective measure to increase the durability of resistance is the pyramiding of 3 or more CR loci in a single cultivar by marker-assisted selection [5]. Our results will enable the development of *Crr1a*-specific or closely linked markers that will improve the efficiency of marker-assisted selection and avoid the introduction of undesirable traits into improved cultivars by linkage drag [40]. Furthermore, knowledge of the pathotype specificity of CR genes is important. *Crr1a^G004^* was effective against pathotypes of groups 2 and 4, but not groups 1 and 3 (Fig. S2). The CR line PL9, with both *Crr1* and *Crr2*, is effective against isolate No. 5 of group 1 [41], and Wakayama-01 distinguished groups 1 and 2 [18], indicating that *Crr2* is useful for resistance to the more virulent isolates. Because *Crr1* comprises *Crr1a* and *Crr1b*, further analysis of the relationships among *Crr1a*, *Crr1b*, and *Crr2* in resistance to different pathotypes is necessary. Recently, Kato et al. [41] showed that *CRb* was effective against isolates of group 3. The pyramiding of *Crr1a^G004^* and/or *Crr1b*, *Crr2*, and *CRb* could confer resistance to most isolates currently present in Japan. In this study, we used field isolates, which are regarded as heterogenic; and multi-pathogenic races have been found in a single field [42], [43]. Single-spore–derived isolates (SSIs) are considered to be most valuable for the genetic study of resistance and virulence surveys of pathogens [43]. The availability of single CR genes and SSIs of *P. brassicae* will allow us to differentiate pathogenicity and pathogen–host interactions more precisely.

## Experimental Procedures

### Plant Materials

A clubroot-resistant (CR) doubled-haploid (DH) line, G004, with *Crr1* and *Crr2*, and a susceptible DH line, A9709, were used as parents for the F_2_ population [19]. To develop a CR inbred line with *Crr1* and *Crr2* in the A9709 background, we selfed one of these CR F_2_ plants to generate a CR F_3_ plant, and backcrossed that line three times to A9709 to generate BC_3_F_3_ plants. During this process, SSR markers linked to *Crr1* and *Crr2* (Suwabe *et al*. 2006) were used to select plants with both loci in each generation. Plants homozygous for the G004 alleles of the *Crr1* and *Crr2* loci were selected from the self progeny of the BC_3_F_3_ plant by SSR genotyping; one of the selected plants was named R4-8-1.

### Test for Clubroot Resistance

The *P. brassicae* field isolates Ano-01, Wakayama-01, and Nos. 5, 7, 9, and 14 were used [12], [13], [18] to test clubroot resistance in *A. thaliana* according to Jubault et al. [35] with modifications: T_2_ seeds were plated on MS medium containing kanamycin to select plants carrying the transgene. Ten-day-old kanamycin-resistant T_2_ plants were transplanted into soil and inoculated by the injection of 2–4 mL of a resting-spore suspension (1.0–1.5×10^6^ spores/mL) into the soil near the roots. Inoculated plants were grown in a controlled environment under 14-h light/10-h dark at 23/18°C. Resistance responses were evaluated 3 to 4 weeks after inoculation, and the disease index (DI) was scored on a scale of 0 to 3 (Fig. S5). The mean DI of each T_1_ line was expressed as the mean of two or three clubroot tests (9 T_2_ seedlings per test) on different dates. Clubroot tests of *B. rapa* were carried out by the insertion method [11], [12]. Each of 2 or 3 tests, performed on different dates, used 8 T_1_ seedlings derived from an independent transgenic *B. rapa* (T_0_). We determined whether the T_1_ plants had the transgene or not by PCR analysis before evaluating resistance. Root symptoms were graded as: 0, no symptoms; 1, very slight swelling on main roots; 2, a small gall on main roots; 2.5, moderate swelling on main roots; 3, severe swelling on main roots (Fig. S4).

### Map-based Cloning of Crr1

A BAC library constructed from the resistant G004 was screened with markers BSA2, AT27, and BSA7 as anchors, and a BAC contig covering 0.6 cM between BSA2 and BSA7 was assembled [3]. We selected F_2_ plants in which recombination occurred in the interval between markers developed from BAC-end sequences. F_3_ plants derived by selfing of the selected F_2_ plants were tested for resistance to Ano-01. To sequence the candidate region for *Crr1a*, we shotgun-sequenced BAC clones, 355H7 and 208F8. Sequences were assembled with Sequencher v. 2 software (Hitachi). ORFs were predicted with Genetyx v. 10 software (Genetyx).

### Vector Construction and Transformation

A 3.7-kb coding sequence of *Crr1a^G004^* was amplified from first-strand cDNA synthesized from root poly(A)^+^ RNA. The *Lactuca sativa Ubiquitin* (*LsUbi*) promoter::*Crr1a^G004^* and *Crr1a^G004^* promoter::*Crr1a^G004^* constructs were generated from the pUC198AAUGU plasmid [44], in which the 1.9-kb *LsUbi* promoter, the *GUS* gene, and the 0.6-kb *LsUbi* terminator were inserted in a modified pUC19 vector, pUC198AA [44]. The amplified *Crr1a^G004^* cDNA replaced *GUS* between the *LsUbi* promoter and terminator in pUC198AAUGU to generate the *LsUbi* promoter::*Crr1a^G004^* construct. A 2.5-kb 5′-upstream sequence of *Crr1a^G004^* replaced the *LsUbi* promoter::*Crr1a^G004^* construct to generate the *Crr1a^G004^* promoter::*Crr1a^G004^* construct. The gene cassettes were ligated into the plant binary vector pZK3B [45]. These constructs were transformed into Arabidopsis Col-0 using *Agrobacterium tumefaciens* strain GV3101 by the floral dip method [46]. For *B. rapa* transformation, a commercial F_1_ cultivar, Osome (Takii Seed Co.), was used as the recipient, and hypocotyl explants were transformed with *A. tumefaciens* strain GV3101 as described [47]. Primer sequences used for vector construction are listed in Table S1.

### GUS Assay Construct and Histochemical Staining

The 2.5-kb 5′-upstream sequence of *Crr1a^G004^* replaced the *LsUbi* promoter of pUC198AAUGU to generate the *Crr1a^G004^* promoter::*GUS* construct. The resulting construct was introduced into Arabidopsis Col-0, and T_2_ plants were used in the reporter gene assay. The GUS assay was carried out according to Ariizumi et al. [48].

### RT-PCR and RACE Analysis

R4-8-1, A9709, and Col-0 plants were inoculated with Ano-01. Total RNA was extracted with a TRIzol Plus RNA Purification Kit (Invitrogen) or an RNeasy Plant Mini Kit (Qiagen) by on-column DNase I treatment, and then converted into first-strand cDNA with a SuperScript III First-Strand Synthesis System for RT-PCR (Invitrogen). Semi-quantitative RT-PCR for *Crr1a^G004^* was performed using cDNA synthesized from RNA isolated from plants at 14 days after inoculation with Ano-01. To test splicing variation, we performed PCR using primer pairs flanking introns 2 and 3 and cloned the PCR products into the pGEM-T Easy vector (Promega) for sequencing. RACE was performed with a BD SMART RACE cDNA Amplification Kit (TaKaRa Bio); 2 independent RACE experiments were carried out. Primer sequences used for these amplifications are listed in Table S1.

### Real-time PCR Analysis

T_1_ seedlings derived from 4 T_0_ lines and wild-type *B. rapa* ‘Osome’ were inoculated with Ano-01, and total RNA was isolated from leaves 4 weeks after inoculation. Transcript levels of the *Crr1a^G004^* transgene were analyzed in 2 independent clubroot tests by real-time PCR using SYBR Premix ExTaq II (TaKaRa Bio). *BrACT2* was used as an internal control for normalization [49]. Primer sequences used for these amplifications are listed in Table S1.

## Supporting Information

Figure S1 **Resistance responses of transgenic Col-0 lines carrying** ***Crr1*** **promoter:** ***Crr1G004*** **cDNA construct (Cp_01–19) or wild-type Col-0 plants, to Ano-01. The mean (±SD) is based on the average of 3 clubroot tests.**
(TIF)

Figure S2 **Resistance of transgenic Col-0 plants with** ***Crr1^G004^*** **to different pathotypes.** Col-0 and 2 T_3_ lines were inoculated with a representative isolate of each pathotype: No. 5 (group 1), No. 7 (group 2), No. 14 (group 3), and No. 9 (group 4). The mean (±SD) is based on the average of 3 tests.
(TIF)

Figure S3 **Schematic representation of** ***Crr1a*** **allelic structure in resistant G004 and susceptible A9709.** Black boxes, exons; black lines, introns; arrowheads, large insertions. White box, *Ty1-copia*-type retrotransposon sequences inserted at 3′-end of exon 4. Sequences of 5′- and 3′-ends of the retrotransposon are shown. Long terminal repeat (LTR) elements (arrows in white box) are underlined in sequences. Putative target site duplication is boxed. Deduced amino acid sequence of *Crr1a^A9709^* is indicated. Asterisk, putative stop codon.
(TIF)

Figure S4 **Typical root symptoms of transgenic** ***Brassica rapa*** **inoculated with Ano-01.** Resistance responses were evaluated 5 weeks after inoculation. Root symptoms were graded as: 0, no symptoms; 1, very slight swelling on main roots; 2, a small gall on main roots; 2.5, moderate swelling on main roots; 3, severe swelling on main roots. Scale bar indicates 5.0 cm.
(TIF)

Figure S5 **Typical root symptoms of Arabidopsis (Col-0) inoculated with** ***P. brassicae*** **.** Resistance responses were evaluated 3 weeks after inoculation. Root symptoms were graded as: 0, no symptoms; 1, very slight swelling on lateral roots; 2, moderate swelling on lateral roots and taproot; 2.5, severe swelling on all roots but no swelling on hypocotyl; 3, severe swelling on all roots and hypocotyl. Scale bar indicates 1.0 cm.
(TIF)

Table S1 Sequences of primers used in this study.
(XLS)
