# Supplementary figures and images for: Identification and Characterization of Crr1a, a Gene for Resistance to Clubroot Disease (Plasmodiophora brassicae Woronin) in Brassica rapa L
Source: PLoS One. 2013 Jan 30;8(1):e54745. doi: 10.1371/journal.pone.0054745 (PMC3559844; doi:10.1371/journal.pone.0054745)

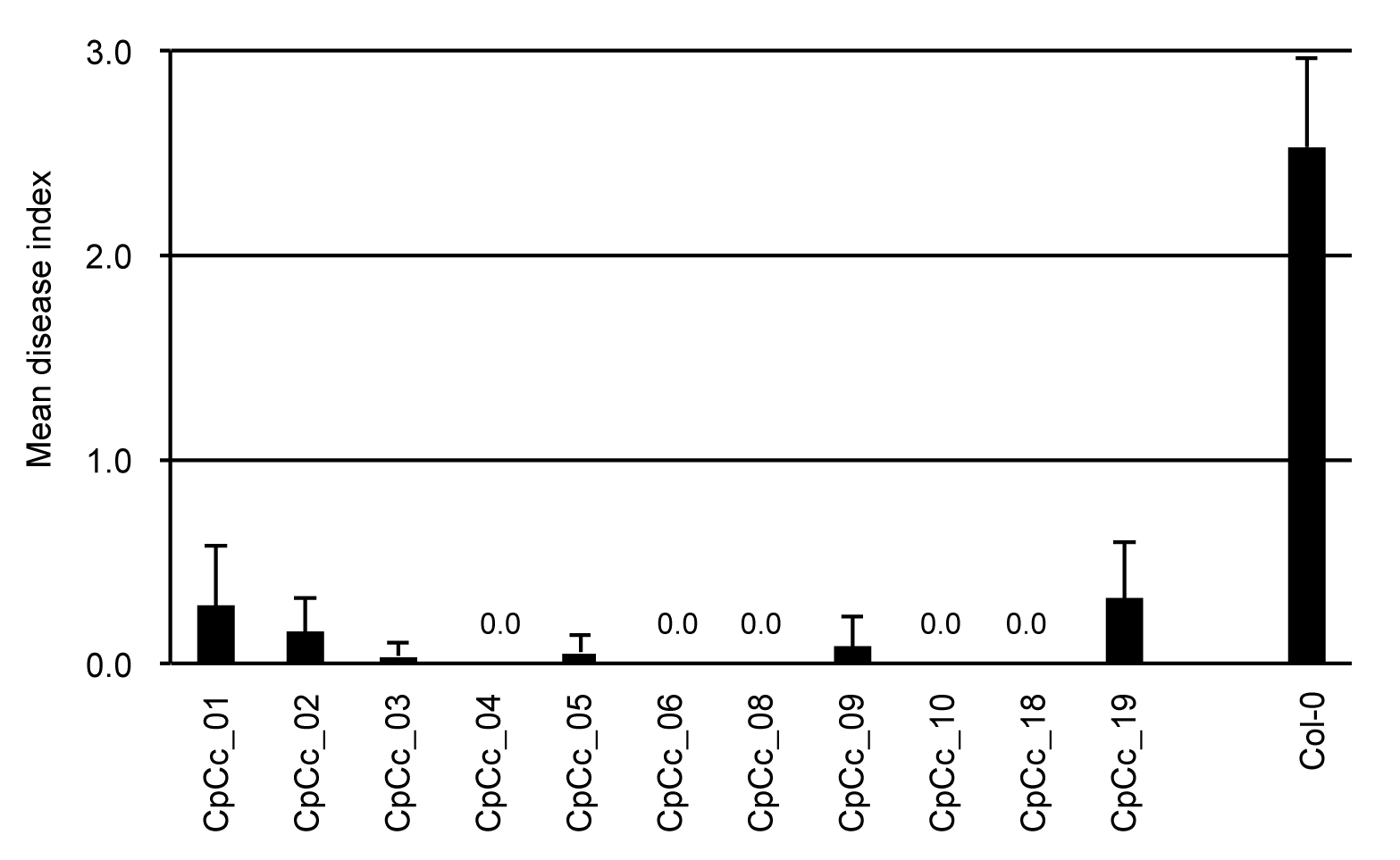

Supplement: Figure S1 — Resistance responses of transgenic Col-0 lines carrying Crr1 promoter: Crr1G004 cDNA construct (Cp_01–19) or wild-type Col-0 plants, to Ano-01. The mean (±SD) is based on the average of 3 clubroot tests. (TIF) [file pone.0054745.s001.tif]

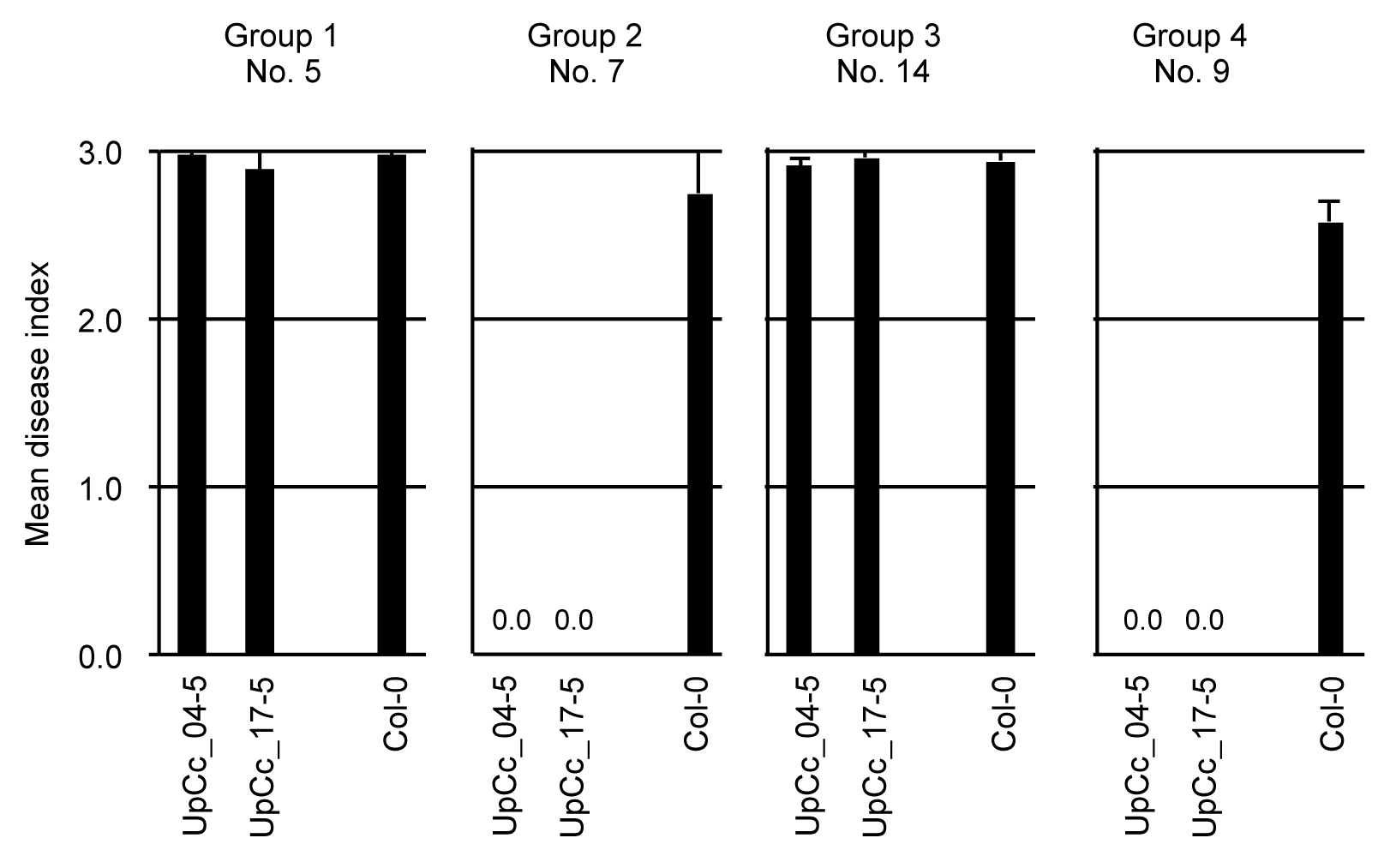

Supplement: Figure S2 — Resistance of transgenic Col-0 plants with Crr1G004 to different pathotypes. Col-0 and 2 T3 lines were inoculated with a representative isolate of each pathotype: No. 5 (group 1), No. 7 (group 2), No. 14 (group 3), and No. 9 (group 4). The mean (±SD) is based on the average of 3 tests. (TIF) [file pone.0054745.s002.tif]

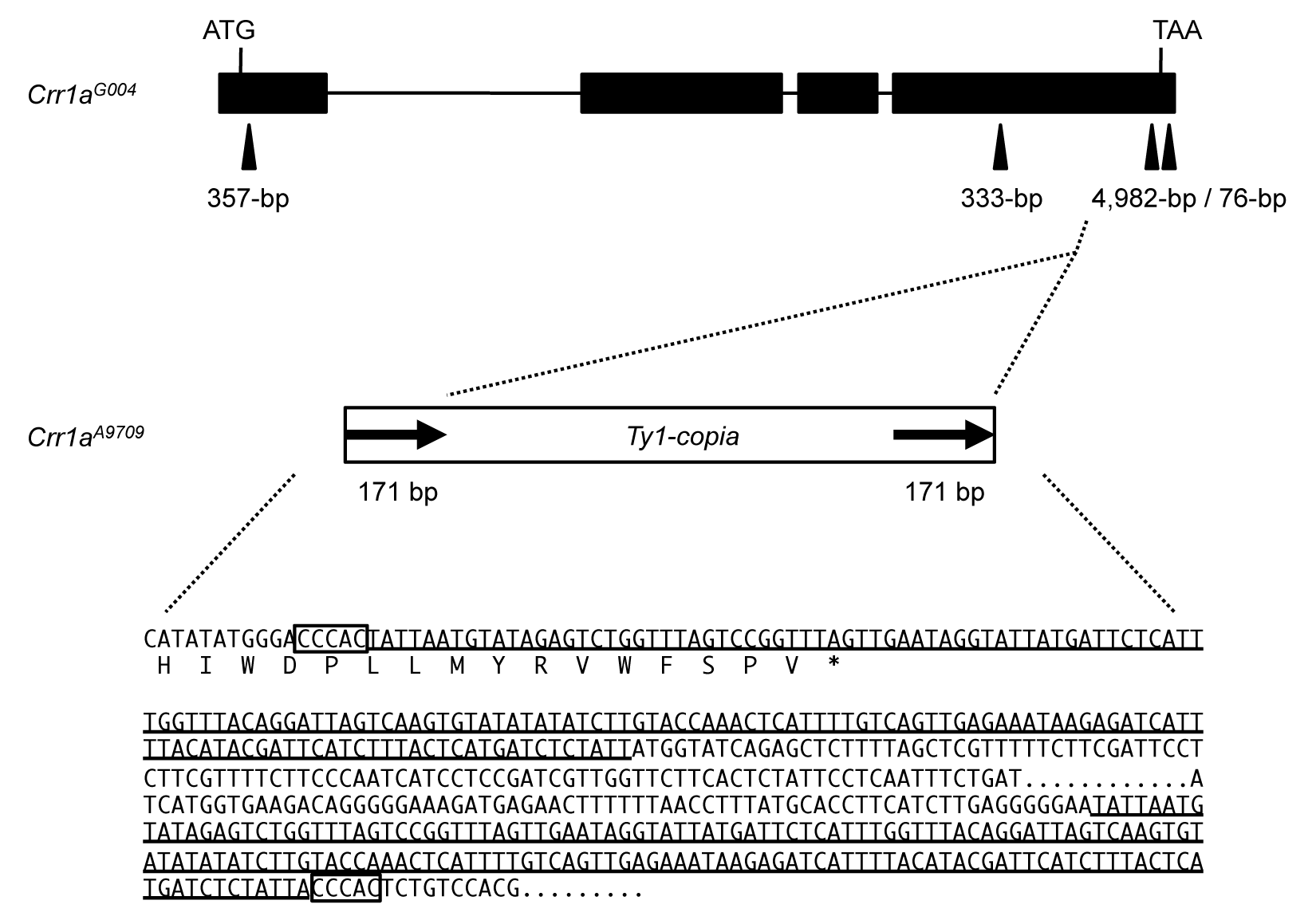

Supplement: Figure S3 — Schematic representation of Crr1a allelic structure in resistant G004 and susceptible A9709. Black boxes, exons; black lines, introns; arrowheads, large insertions. White box, Ty1-copia-type retrotransposon sequences inserted at 3′-end of exon 4. Sequences of 5′- and 3′-ends of the retrotransposon are shown. Long terminal repeat (LTR) elements (arrows in white box) are underlined in sequences. Putative target site duplication is boxed. Deduced amino acid sequence of Crr1aA9709 is indicated. Asterisk, putative stop codon. (TIF) [file pone.0054745.s003.tif]

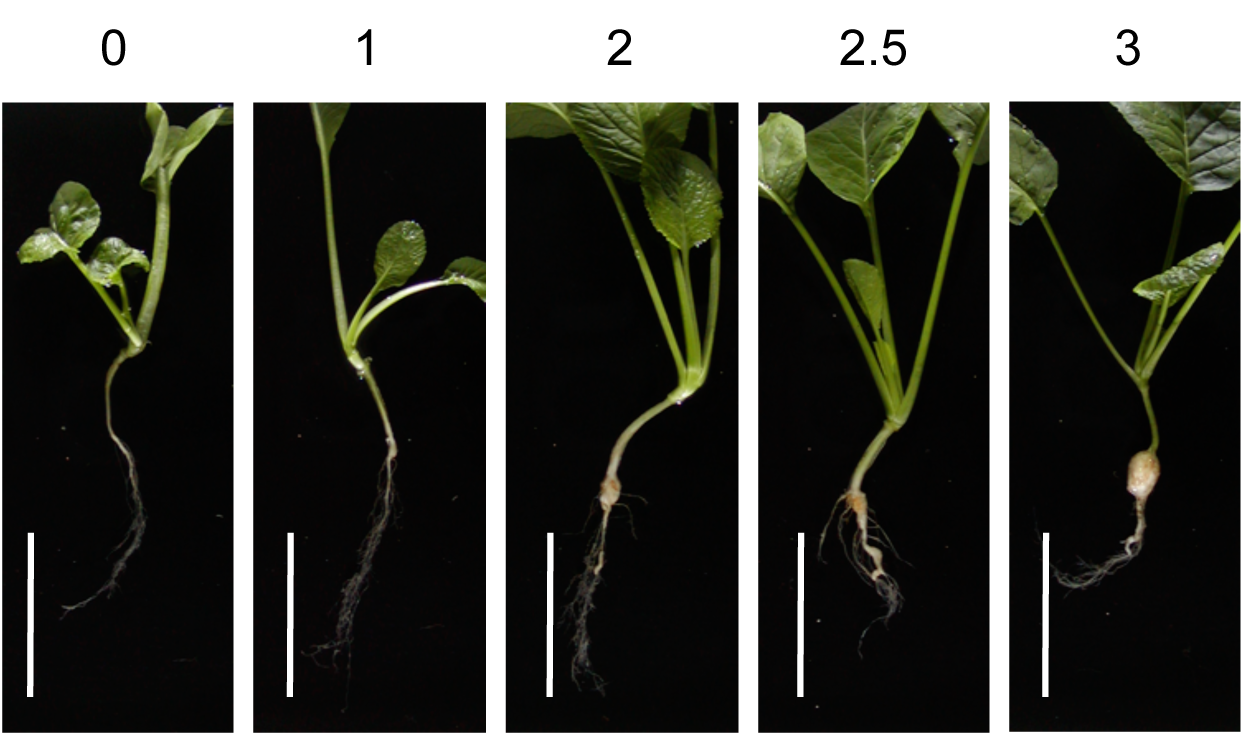

Supplement: Figure S4 — Typical root symptoms of transgenic Brassica rapa inoculated with Ano-01. Resistance responses were evaluated 5 weeks after inoculation. Root symptoms were graded as: 0, no symptoms; 1, very slight swelling on main roots; 2, a small gall on main roots; 2.5, moderate swelling on main roots; 3, severe swelling on main roots. Scale bar indicates 5.0 cm. (TIF) [file pone.0054745.s004.tif]

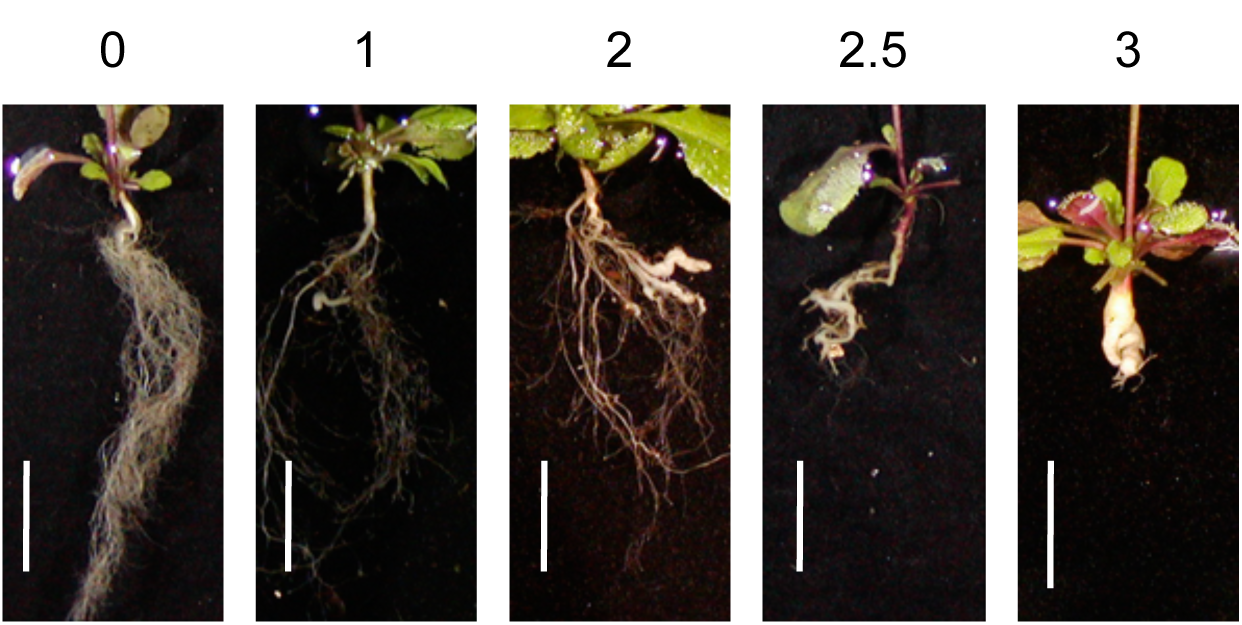

Supplement: Figure S5 — Typical root symptoms of Arabidopsis (Col-0) inoculated with P. brassicae . Resistance responses were evaluated 3 weeks after inoculation. Root symptoms were graded as: 0, no symptoms; 1, very slight swelling on lateral roots; 2, moderate swelling on lateral roots and taproot; 2.5, severe swelling on all roots but no swelling on hypocotyl; 3, severe swelling on all roots and hypocotyl. Scale bar indicates 1.0 cm. (TIF) [file pone.0054745.s005.tif]
